# Street youths: reproductive health risk status, reproductive health challenges and barriers to health services utilization in a southwestern City, Nigeria

**DOI:** 10.4314/ahs.v22i3.7

**Published:** 2022-09

**Authors:** Adenike Iyanuoluwa Olugbenga-Bello, Oluwatosin Ruth Ilori, Tokunbo Idowu

**Affiliations:** 1 Ladoke Akintola University of Technology College of Health Sciences, Community Medicine; 2 Ladoke Akintola University of Technology Postgraduate School, Community Medicine

**Keywords:** street, youths, reproductive health, Ikorodu

## Abstract

**Background Information:**

According to the United Nations, about 150 million youth spent most of their time on the street, or better still, homeless. This is becoming a global phenomenon and majority of this vulnerable people live in large cities and urban areas of developing countries. Street youths are among the high risk, insecure and vulnerable groups who are often exposedto various forms of abuses and diseases, including reproductive health issues.

**Methodology:**

A descriptive cross sectional study carried out among street youths in Ikorodu Local Government, Lagos State using a multi staged sampling technique. Frequency tables were drawn at the univariate level, chi squared was used to test for association between socio-demographic characteristics and sexual risk level. Data was analyzed using SPPSS version 22, p value was set at 0.05

**Results:**

Almost half 48(48.5%) of the respondents were between the age range 20–24years and two third 61(61.6%) of them were female and 27(27.3%) had up to senior secondary education attainments. Majority 73(73.7%) of them have been on the street for more than 3months and 32 (32.3%) professed that the reason they were on the street was to search for job while 25 (25.3%) because of family disharmony among parents. Eighty six (86.9%) of the respondents were sexually active, 31 (36.0%) of which have more than four sexual partners. Duration of stay on the street and their educational status were determinants of risky sexual behavior and polygamous setting was found to be statistically significant(p value =0.035) with reproductive health challenges.

**Conclusion:**

There is high risk sexual practice among street youths in Ikorodu Local Government. Strategic interventions aimed at minimizing sexual risky behaviors among street youths should focus on reducing the duration of stay on the street as well as increasing access to contraception.

## Introduction

The phenomenon of homeless/street children is global, with the streets throughout the world being home to millions of children. Tens of millions of children are living or working on the world's streets. This number keeps growing due to population growth, intensifying urbanization and migration, particularly in the developing world, amongst others.^1^

According to UNICEF and other actively engaged NGOs, there are approximately 500,000–700,000 street youths nationally, and an additional 1 million are at risk for streetism.^2^

Street youths are exposed to situations that make them vulnerable to sexual and reproductive health problems on a day to day basis, as they often than not engage themselves in risky sexual behaviors.^3^,^4^ A risky sexual behavior is one that increases the likelihood of adverse sexual and reproductive health consequences.^5^ Available data shows that HIV sero-prevalence rates for street children are 10–25 times higher than non-street adolescents.^6^,^7^ This is because street children are reported to become sexually active earlier than the other groups of adolescents.^5^,^7^ They also engage in sex with many sexual partners and are likely to be coerced into sexual relationships to ensure their survival. They use condoms inconsistently and get inadequate information about sexuality.^5^–^8^

Street youths are predisposed to sexual and reproductive health challenges. Most of the street children live in severe deprivation, which make them liable to various forms of health risks. Street youths have risky sexual behaviours that increase the likelihood of adverse sexual and reproductive health consequences.^5^

Children are pushed into living and working on the street by many factors such as natural disasters, wars, poverty, economic recessions, domestic abuse and family crises, or even the ideal of freedom that is thought to be found on the streets are all among the reasons causing boys and girls, young men and women to seek means for survival^10^, and many times, they take to street life in order to relieve parents of burden of care. Once on the streets many other threats await these children; some of the most pressing challenges street children face include difficulties in maintaining basic health and accessing health services, violence and abuse, and dangerous working conditions which makes them vulnerable to various kinds of psychological problems and health risks.^10^

Basic needs for money, food, shelter and other essential necessities of life co-join with early initiation of sexual activity^10^ place street children at high risk of becoming involved in the sex trade or of practicing ‘survival sex’ in exchange for food, shelter, money or drug. Substance use also increases the likelihood that individuals will engage in risky sexual behaviours, for example, non-condom use and multiple sexual partner ptterns which put them at risk of sexually transmitted infections (STIs) and HIV/AIDS. In spite of the visibility of the street youths, they have limited access to basic services, such as quality health care services, basic social amenities etc. and they often face challenges if peradventure they were able to access healthcare such as cost of care, stigmatization by providers, distrust in quality of care, and difficulty finding time to seek care because of lost or meager earnings, unsafe sex and unintended pregnancy.

The objective of this study was to identify reproductive health challenges as well as risky sexual behaviors of street youths in Ikorodu.

## Methodology

### Description of study area

Ikorodu LGA is one of the largest in Lagos State. It covers a land area of about 161.954km with an East /West diameter of about 15km. Ikorodu Local Government has thirty (30) political wards with a projected population of 822,847 in year 2019, among which 32,914 are children 0–11months (4%), 164,569 are children 0–59 months (20%), 181,026 are women of child bearing age(22%), while 41,142 are pregnant women(5%). Men are 403,195 and 419,652 are women. There are 29 (twenty nine) public Primary Health Centers, two (2) General Hospitals, and one hundred and eighty two (182) Private Health Care facilities. Ikorodu General Hospital is located in a very central and accessible area, opposite the Local Government Council secretariat while the second General Hospital is located at Ijede LCDA

### Study population

These were street youths within the ages of 18 and 36 years in Ikorodu Local Government. They involved both the youths on the street and the youths of the street. The former have a family tie and go back home at the end of the day while the latter live their lives entirely on the street. A total sampling was done.

### Study design

This was a descriptive cross-sectional study.

### Sampling technique

Six wards were selected from the thirty political wards in Ikorodu Local Government using simple random sampling (Balloting technique). Two settlements were selected each from the six chosen wards selected using simple random sampling (Balloting technique) making a total of twelve settlements. All street youth in those settlements were sampled.

### Research Instruments

A semi-structured interviewer administered questionnaire was used, adapted from various articles. Research assistants were used for the administration of the research tool after being trained in order to prevent ambiguity of meaning. The questionnaire was divided into five sections namely a, b, c, d and e. The questionnaire researched into the socio-demographic characteristics, knowledge of reproductive health issues, risky sexual practices of respondents. The questionnaire was translated to Yoruba Language and later back translated to English Language in order to ensure that the original meaning was not distorted.

### Data Analysis

Data was collected and analyzed using SPSS version 22. Frequency tables were drawn at the univariate level while Chi square was used to test for association between socio-demographics and risky sexual behaviors of respondents. At the multivariate level, logistic regression was done to extract determinants of risky sexual behavior among street youths. Level of significance was put at p>0.05

### Scoring system

Respondents who had sex without condom and those who engages in night parties were considered to practice risky sexual behavior. Risky behaviors were scored 1 while non-risky behaviors were scored 0. The overall score for each respondent was summed up and the mean gotten. All the respondents with score above the mean were regarded to have high risky sexual exposure while respondents with scores below the mean were regarded to have low risk sexual exposure.

### Response rate

One hundred (100) research instruments were distributed to the respondents and ninety-nine (99) were retrieved, giving a response rate of 99.0%.

## Results

**Table 1:** Showed the socio-demographic characteristics of respondents. It showed that closed to half 48(48.5%) of the respondents were between the age range 20–24years and two third 61(61.6%) of them were female while 27(27.3%) had up to senior secondary education attainments. Half 49(49.5%) of them were Muslims and 69(69.7%) belongs to Yoruba ethnic group while 32(32.3%) said they were on the street because they were looking for the job. Majority 73(73.7%) of them have been on the street for the past 3months out of which 68(68.7%) go home at the close of the day. The research shows that 52(52.5%) of the respondents were from polygamous families while 41(41.4%) were from monogamous family background. About one third 29(29.3%) of them were fourth /fifth born in their family and 51(51.5%) said their parents were married while one third 33(33.3%) said their parents were divorced.

**Table 1:** Socio-demographic characteristics of respondents (N=99)

Variables	Frequency (n)	Percentage(%)
**Age (years)** 0–14 15 – 19 20 – 24	4 47 48	4.0 47.5 48.5
**Gender** Male Female	61 38	61.6 38.4
**Educational Qualification** Primary school Cert JSCE SSCE OND, NCE or equivalent No response	26 12 27 17 17	26.3 12.1 27.3 17.2 17.2
**Religion** Christianity Islam Traditional	49 42 8	49.5 42.4 8.1
**Ethnicity** Yoruba Hausa Ibo No response	69 13 5 12	69.7 13.1 5.1 12.1
**Reasons for being on the street** Looking for a job Poverty Family disharmony Death of parents Peer pressure Others	32 20 25 5 11 6	32.3 20.2 25.3 5.1 11.1 6.1
**Duration of been on the street** Less than 3 months Greater than 3 months	26 73	26.3 73.7
**Type of street life** On the street Of the street	68 31	68.7 31.3
**Family type** Monogamy Polygamy Polyandry	41 52 6	41.4 52.5 6.1
**Birth Order in the family** First Second Third Others	21 23 26 29	21.2 23.2 26.3 29.3
**Parents Marital Status** Single Parent Married Divorced	33 10 5	33.3 10.1 5.1

**Table 2:** Showed the Sexual risk behaviour practice among respondents. It shows that 86(86.9%) of the respondents ever had sexual intercourse and 41(47.7%) of them had their first sexual intercourse at age 15–19years old while 62(72.1%) of them said they had straight sex orientation. More than half 57(66.3%) of those that ever had sex, said they usually engaged in vagina sexual intercourse and 34(38.8%) claimed both drugs and alcohol as their sexual stimulant while 31(36.0%) had more than four sexual partners. More than half 56(56.6%) of the respondents ever used condom in their life time and 31(55.4%) of those that used condom usually use it occasionally while 8(14.3%) rarely use condom. Only 24(24.2%) of them ever used contraceptive method and 54(54.5%) said they know means of avoiding pregnancy while 12(50.0%) of those that are on contraceptive claimed condom as the one they used. Half 31(50.5%) of the males said they had sex with the commercial sex workers in that last 3 months while 23(60.5%) of the female ever had sex with commercial sex workers in last 3 months.

**Table 2:** Sexual risk behaviour practice among respondents (N=99)

Variables	Frequency (n)	Percentage(%)
**Ever had sexual intercourse** Yes No	86 13	86.9 13.1
**Age of first sexual experience** 10–14 15–19 20–24	n=86 26 41 19	30.2 47.7 22.1
**Sexual orientation** Homosexual Lesbian Heterosexual	n=86 16 8 62	18.6 9.3 72.1
**Type of sex usually engage in** Anal Vagina Both	n=86 2 57 27	2.3 66.3 31.4
**Number of sexual partner** 1 2 3 >4	26 16 13 31	30.2 18.6 15.1 36.0
**Ever had sex with commercial sex workers in last 3months(Male)** Yes No	n=61 31 30	50.5 49.5
**Ever had sex with commercial sex workers in last 3months (female)** Yes No	n=38 23 15	60.5 39.5
**Frequency of condom use** Occasionally Regularly Rarely	n=56 31 17 8	55.4 30.4 14.3
**Ever use modern contraceptive method** Yes No	24 75	24.2 75.8
**Types of contraceptive used** Condom Injectable Implants	n=24 12 8 4	50.0 33.3 16.6
**Reproductive Health Problems** Unwanted Pregnancy Abortion Rape STI	10 14 15 42	10.2 14.1 15.2 42.9
Risky behaviors* Unprotected sex Drinking of alcohol Smoking Use of hard drugs	86 70 51 64	86.9 70.7 51.5 64.3
Kinds of drug used* Maurijuana Cigarette Tramadol Codeine Weed	n=64 8 24 14 11 18	15.7 47.1 31.4 35.3 11.4

**Table 3:** Showed the reproductive Health Challenges experienced by respondents. It shows that more than half 55(55.6%) of the respondents ever visited health institution and the major reason for their visit were for HIV/AIDs counseling, for sexual transmitted infection, for uptake of contraceptive and other reasons best known to them while the major health institution visited was public health institution (61.8%). More than half 54(54.5%) ever had skin disorders and 27(27.3%) have ever been harassed by the gangsters while 38(38.4%) haveever been stigmatized by the people around them. Few 4(4.0%) of the respondents have ever been jailed before only 27(27.3%) had good three square meal daily.

**Table 3:** Social and Reproductive Health Challenges of respondents (N=99)

Variables	Frequency (n)	Percentage(%)
**Ever visited health institution** Yes No	55 44	55.6 44.4
Reasons for visit* Had STIs For contraceptives For abortion For delivery HIV/AIDS counseling Others	n=55 23 19 7 11 26 32	40.0 34.5 12.7 20.0 47.3 58.2
**Health institution visited** Pharmacy Private health sector Public health institution Family Guidance clinic	n=55 15 4 34 2	27.3 7.3 61.8 3.6
**Health challenges** Ever had any skin disorders Sexually abused Infested with lice	54 45 38	54.5 45.5 38.4
**Social challenges** Harassed by Police Harassed by gangsters Access to schooling Stigmatized by people Ever been without clothing Ever been jailed before Have a source of livelihood Eat three square meal daily	42 27 35 38 47 4 36 27	42.4 27.3 35.4 38.4 47.5 4.0 36.4 27.3

**Table 4:** Showed the respondents recommendation to the concerned bodies, it shows that 31(31.9%) stated that government should provide avoidable housing for the homeless while 5(5.2%) said that government and concerns bodies should create micro credit agency for the young once to have access to loan.

**Table 4:** Respondents recommendation to the concerned bodies

Variables	Frequency	Percentage
Government should provide affordable housing for the homeless	31	31.9
More skills acquisition program to empower the children on the street	19	19.6
More social program that will expose the street adolescents to sex education	26	26.8
The government should provide more job opportunity for the young ones	16	16.5
Creation of micro credit agency for the young once to have access to loan	5	5.2

**Table 5:** Showed the association between socio-demographic characteristics and sexual risk behaviour practice. It shows that shows that source of income and duration of being on the street were statistically significant with respondents with their sexual risk behaviour practice with pvalue<0.05.

**Table 5:** Association between socio-demographic characteristics and risk status category

Variables	Risk Category		Total	Statistics
	High risk (n=62)	Low risk (n=37)		
**Age (years)** 10–14 15 – 19 20 – 24	3(75.0) 31(66.0) 28(58.3)	1(25.0) 16(34.0) 20(41.7)	4(100.0) 47(100.0) 48(100.0)	X^2^=0.456 df=2 Pvalue=
**Gender** Male Female	37(60.7) 25(65.8)	24(39.3) 13(34.2)	61(100.0) 38(100.0)	X^2^=0.264 df=1 Pvalue=0.673
**Educational** **Qualification** Primary school Cert JSCE SSCE OND, NCE or equivalent No formal education	18(69.2) 8(66.7) 17(63.0) 11(64.7) 8(47.1)	8(30.8) 4(33.3) 10(37.0) 6(35.3) 9(52.9)	26(100.0) 12(100.0) 27(100.0) 17(100.0) 17(100.0)	X^2^=2.361 df=4 Pvalue=0.670
**Religion** Christianity Islam Traditional	32(65.3) 24(57.1)	17(34.7) 18(42.9)	49(100.0) 42(100.0)	X^2^=1.213 df=1 Pvalue=0.545
**Ethnicity** Yoruba Hausa Ibo Others	41(59.4) 8(61.5) 5(100.0)	28(40.6) 5(38.5) 0(0.0)	69(100.0) 13(100.0) 5(100.0) 12(100.0)	X^2^=3.377 df=3 Pvalue=0.337
**Source of income** Gambling Manual labour Trading	17(85.0) 33(55.9) 12(60.0)	3(15.0) 26(44.1) 8(40.0)	20(100.0) 59(100.0) 20(100.0)	X^2^=7.466 df=2 Pvalue=0.045*
**Reasons on the street** Looking for a job Poverty Family disharmony Death of parents Peer pressure Others	19(59.4) 10(50.0) 14(56.0) 4(80.0) 11(100.0) 4(66.7)	13(40.6) 10(50.0) 11(44.0) 1(20.0) 0(0.0) 2(33.3)	32(100.0) 20(100.0) 25(100.0) 5(100.0) 11(100.0) 6(100.0)	X^2^=9.227 df=5 Pvalue=0.100
**Duration on the street** Less than 3 months Greater than 3 months	15(57.7) 47(64.4)	11(42.3) 26(35.6)	26(100.0) 73(100.0)	X^2^=10.367 df=1 Pvalue=0.035*
**Type of street life** On the street Of the street	44(64.7) 18(58.1)	24(35.3) 13(41.9)	68(100.0) 31(100.0)	X^2^=0.401 df=1 Pvalue=0.339

* Statistically significant <0.05

**Table 6:** showed the association between socio-demographic characteristics and respondents level of exposure to RH challenges. It shows that more of respondents with low sexual risk behavior are less exposed to reproductive health challenges compared those with high sexual behaviors, which was statistically significant with pvalue 0.005.

**Table 6:** Association between socio-demographic characteristics and Reproductive Health challenges categories

Variables	Overall exposure to RH challenges		Total	Statistics
	Low exposure(n=58)	High exposure(n=41)		
**Age (years)** 10–14 15 – 19 20 – 24	3(75.0) 31(66.0) 24(50.0)	1(25.0) 16(34.0) 24(50.0)	4(100.0) 47(100.0) 48(100.0)	X^2^=2.955 df=2 Pvalue=0.228
**Gender** Male Female	38(62.3) 20(52.6)	23(37.7) 18(47.4)	61(100.0) 38(100.0)	X^2^=0.901 df=1 Pvalue=0.404
**Educational Qualification** Primary school Cert JSCE SSCE OND, NCE or equivalent No formal education	16(61.5) 8(66.7) 17(63.0) 6(35.3) 11(64.7)	10(38.5) 4(33.3) 10(37.0) 11(64.7) 6(35.3)	26(100.0) 12(100.0) 27(100.0) 17(100.0) 17(100.0)	X^2^=17.693 df=4 Pvalue=0.032*
**Religion** Christianity Islam Traditional	26(53.1) 25(59.5) 7(87.5)	23(46.9) 17(40.5) 1(12.5)	49(100.0) 42(100.0) 8(100.0)	X^2^=3.388 df=2 Pvalue=0.184
**Ethnicity** Yoruba Hausa Ibo Others	36(52.2) 11(84.6) 4(80.0) 7(58.3)	33(47.8) 2(15.4) 1(20.0) 5(41.7)	69(100.0) 13(100.0) 5(100.0) 12(100.0)	X^2^=5.745 df=3 Pvalue=0.125
**Source of income** Gambling Manual labour Trading	13(65.0) 37(62.7) 8(40.0)	7(35.0) 22(37.3) 12(60.0)	20(100.0) 59(100.0) 20(100.0)	X^2^=3.601 df=2 Pvalue=0.165
**Reasons for being on the** **street** Looking for a job Poverty Family disharmony Death of parents Peer pressure Others	17(53.1) 10(50.0) 12(48.0) 3(60.0) 10(90.0) 6(100.0)	15(46.9) 10(50.0) 13(52.0) 2(40.0) 1(9.1) 0(0.0)	32(100.0) 20(100.0) 25(100.0) 5(100.0) 11(100.0) 6(100.0)	X^2^=11.138 df=5 Pvalue=0.049*
**Duration of been on the** **street** Less than 3 months Greater than 3 months	17(65.4) 41(56.2)	9(34.6) 32(43.8)	26(100.0) 73(100.0)	X^2^=6.672 df=1 Pvalue=0.028*
**Type of street life** On the street Of the street	42(61.8) 16(51.6)	26(38.2) 15(48.4)	68(100.0) 31(100.0)	X^2^=0.904 df=1 Pvalue=0.232

* Statistically significant <0.05

**Table 7:** Showed the predictors of sexual risk practice among current users of family planning among respondent. None of the predictors were statistically significant.

**Table 7:** Predictors of sexual risk practice among respondents

Explanatory factors	B	OR (95% CI)	df (p-value)
**Educational status**			
Educated (Ref)			
Not educated	-0.189	0.828(0.319 – 2.149)	1(0.698)

**Source of income**			
Gambling (Ref)			
Manual labour	-1.399	0.247(0.052 – 1.172)	1(0.078)
Trading	0.090	1.094(0.376 – 3.186)	1(0.869)

**Duration on the street**			
Less than 3 months(Ref)			
Greater than 3 months	0.077	1.080(0.368 – 3.170)	1(0.889)

**Family type**			
Monogamy(Ref)			
Polygamy	1.447	4.250(0.424 – 42.622)	1(0.219)
Polyandry	1.075	2.931(0.269 – 31.950)	1(0.378)

Omnibus test; X^2^ = 8.447, p-value 0.207, Correct classification percentage; 62.6%

**Table 8:** There is a statistical association between family type and reproductive health challenges of respondents. None of the predictors were statistically significant

**Table 8:** Predictors of exposure to reproductive health challenges among respondents

Explanatory factors	B	OR (95% CI)	df (p-value)
**Educational status**			
Educated (Ref)			
Not educated	0.210	1.234(0.407–3.736)	1(0.710)

**Source of income**			
Gambling (Ref)			
Manual labour	0.209	1.232(0.376–4.043)	1(0.731) 1(0.153)
Trading	1.187	3.278(0.642–16.727)	

**Duration on the street**			
Less than 3 months(Ref)			
Greater than 3 months	0.908	2.479(0.673 – 9.130)	1(0.172)

**Family type**			
Monogamy(Ref)			
Polygamy	-1.170	0.310(0.104–0.925)	1(0.036)*
Polyandry	-0.893	0.409(0.047 – 3.593)	1(0.420)

**Reasons for been on the street**			
Looking for a job			
Poverty	0.082	1.086(0.311 – 3.787)	1(0.897)
Family disharmony	0.140	1.150(0.334 – 3.963)	1(0.825)
Death of parents	-1.333	0.264(0.026 – 2.720)	1(0.263)
Peer pressure	-2.108	0.121(0.012 – 1.209)	1(0.072)
Others	-21.184	0.000	1(0.999)

Omnibus test; X^2^ = 21.781, p-value=0.026, Correct classification percentage; 66.7%

**Figure 1:**
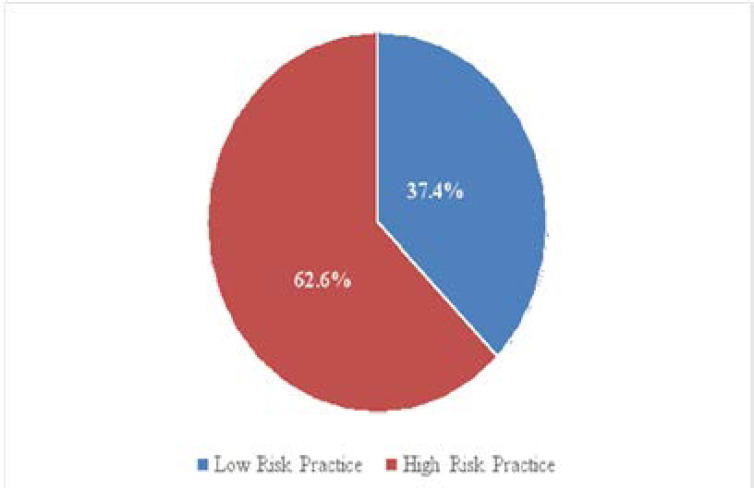
Risk status of respondents

**Figure 2:**
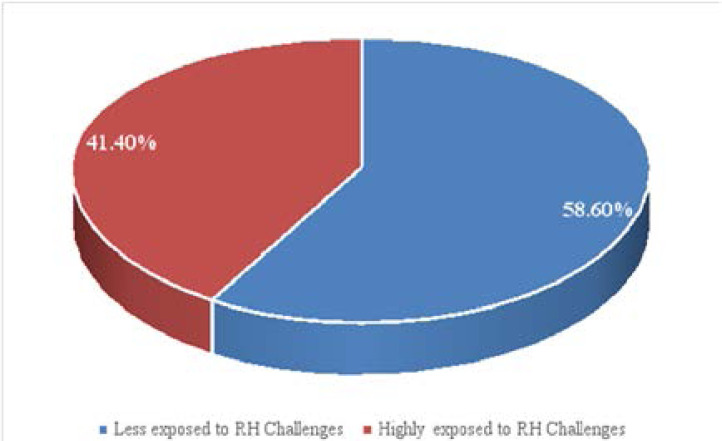
Respondents category of challenge

**Figure 1:** Showed that two third 62% of the respondents had high risk sexual behaviour practice while 37% had low risk sexual behaviour practice;

**Figure 2:** Showed the overall reproductive health challenges experienced by respondents. It shows that 58.6% of the respondents were highly exposed to reproductive health challenges.

**Figure 3:**
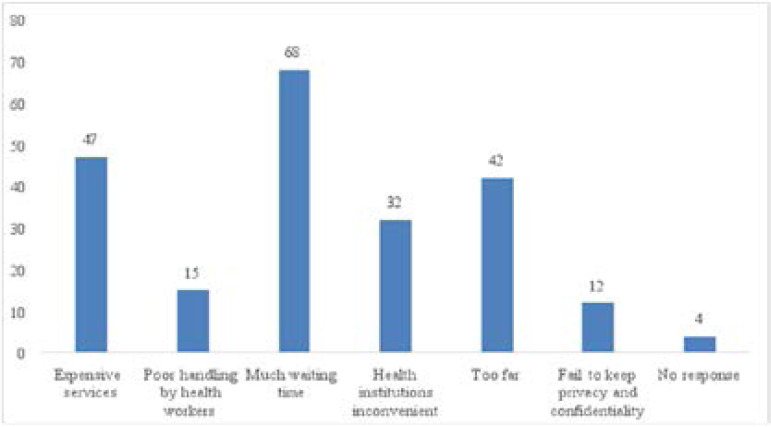
Respondents view on barriers to utilization of health services *Statistically significant <0.05

Figure 3: Showed the respondents view on barriers to utilization of health services. It shows that sixty-eight (68) of the respondents claimed to much waiting time as the barrier for visiting health facilities while fifteen (15) of them claimed poor handling by health workers.

## Discussion

In this study, the main reason for being on the street was family disharmony and the bid to search for job. This is not farfetched from the result of a systematic review of a hundred and eight countries where family conflict as well as changes in the family structure such as death of either or both parents were identified as causes of streetism among respondents.^11^ The need for family harmony in putting an end to this terrible menace of streetism cannot be over emphasized. When parents cannot fend adequately for their children, the latter may look for other means to get their desires met. Most of them resort to the street to get their daily and essential needs met. The need to increase the economic status of families in the communities should be advocated, this will help parents rise up to the unlimited need that stare at their children's face on a daily basis. In addition, it will probably also reduce the number of children on the street to the barest minimum. On the contrary, however, a qualitative study done in Ethiopia showed that the death of parents forced youths and children to the street.^12^

A review done on the effect of polygamy revealed that children and adolescents from polygamous families seem to have more mental health challenges, social problems and lower academic performance than those from monogamous families.^13^ In this current study, more than half of respondents were from a polygamous family and a higher percentage of them were fourth born and more. Being born in a polygamous family was found to be statistically significant with sexual reproductive health challenge at the multivariate level. The probable reason for this is the likelihood of been neglected by parents especially in homes with many children, which is more likely in a polygamous setting. The father may not be able to bear the responsibility of the children if they are too many for his economical limit. Such children may likely find refuge on the street to help them meet their unlimited daily needs.

Six out of ten of the sexually active respondents were involved in heterosexual sex, mostly vaginal. In addition, almost four-tenth of them have more than four sexual partners which make them more vulnerable to sexually transmitted diseases. Surprisingly, despite the high rate of sexual partners, more than half of the respondents use condom occasionally. This finding is in line with another study on the sexual behavior of street youths where it was revealed that majority of the sexually active young adults had several sexual partners but very few of them used condom.^3^ A similar event was observed in Ethiopia where participants reported inconsistent and non use of condom during intercourse.^12^ This is detrimental to the health of such individuals because they are known to have a high risk of sexually transmitted infections and also stand the risk of unwanted pregnancies.

A less exposure time to the streets invariably means a less contact with the street culture, peer pressure and, consequently, sexual risky behaviors. It is also suggested that spending less time on the streets during the day may be associated with spending more time at school or under the supervision of social support organizations, thus decreasing the time of exposure to peers that can lure into anti social behavior.^14^ Data from this study revealed that more than two third of respondents had been on the street for more than three months. In fact, every six out of ten of them live most of their life on the street with no family tie. This is quite disheartening and has the tendency to expose them to more social and health challenges if a definite solution is not provided as soon as possible.

Furthermore, more than two third of the respondents have ever had sexual intercourse, half of which were within the age range of 15–19 years. The number is quite higher than that obtained from another study done on the determinants of sexual and reproductive Health among Street Adolescents where it was discovered that half of them were involved in high risk sexual behavior.^15^ In addition, only two tenth of the street youths have ever used a form of contraceptive method during sexual intercourse. That is also in tandem with a study done in India where majority of the respondents failed to use condom during sex. 15 Another study done in the Republic of Congo on the risk behavior of street adolescents however revealed that more than half of the respondents used condom during intercourse.^16^ A probable reason for this difference may be in the study location as the later study was done among street youths under rehabilitation. They possibly had better access to reproductive health information when compared with their counterparts who are still on the street. Another reason adduced to this may be the non existing Adolescent Reproductive health services targeted towards the well being of this vulnerable youths in Nigeria.

Furthermore, more than two third of respondents in this study take alcohol, and a little above half smoke. However, in another study done in Brazil on drug use among street children and adolescents, tobacco and inhalants topped the list of the substances abused among the participants.^14^ A probable reason for this could be that more of the substances are available in Brazil compared to Nigeria.

About half of the respondents have ever visited a health institution and a higher percentage of them were there to seek counseling on HIV/AIDS prevention. This may be a pointer to the fact that they were aware of the likely diseases they were exposed to.

Only about one fifth of the respondents have access to three square meal on a daily basis. That has the propensity of adversely affecting their nutrition and invariably making them more vulnerable to diseases that could have been prevented with adequate diet. More of those whose source of income was gambling had tendencies towards risky behaviors which was statistically significant. In addition, more of the respondents who had spent more than three months on the street have a higher and statistically significant tendency for risky sexual behavior compared with those who have spent less than three months. In other words, the longer a youth stays on the street, the more risky behaviors he/she exhibits. This may be due to peer group influence and the absence of adequate and timely parental guidance.

Of note, it was found out that more than three fifth of the respondents from a polygamous family were engaged in high risk practices compared with those from monogamous settings, which was statistically significant. It's possible they felt neglected since there were other children to be catered for at home. That may also be the reason why they were out on the street in the first place.

Education also tends to determine how exposed an individual would be to reproductive health challenges. Respondents with lower educational status tend to be less exposed to RH challenges compared with those with higher educational qualification which was statistically significant. The probable reason for this may be the higher exposure to peers and social media by those in the higher places of learning. They are more likely to have access to social devices like mobile phones, and the internet which has a whole lot of activities that can tilt them towards risky sexual behaviors. Furthermore, they may have more money to procure drugs and probably also pay for sex compared with those with lower educational status.

Six out of 10 of the studied adolescents were highly exposed to sexual risk behavior while one third of them were minimally exposed to sexual risk practices. The adolescents should be made aware of the risky sexual behaviours that increase the likelihood of adverse sexual and reproductive health consequences by continuous education through both the mass and social media through both Governmental and Non-Governmental bodies. Another research done which was in contrast with this study showed that more out-of-school youths in Nigeria participate frequently in risky sexual behaviors than their in-school counterparts. It is also in agreement with a study by Olle y^7^ where sexual risk behavior was linked to poverty, early marriage, or other vulnerable situations such as being from broken homes or living on the streets.^1^

Predictors of risky behavior in a study by Kamanu includes male gender and age in years.^15^ In this study, however, the source of income as well as the duration of stay on the street were found to be the determinants of risky behavior among the respondents. In another study done on the cross national Variations in behavioral profiles among homeless youths, it was found that more than 30% of respondents have been pregnant before. The figure is however higher than that found in this study where barely less than two fifth of respondents have had unwanted pregnancy at one time or the other.

According to this study, expensive services as well as long waiting time were mostly mentioned as reasons why youths don't access reproductive health services. There is a need to make the available services more youth friendly by subsidizing services as well as giving them priority when they visit health facilities.

The implication of poor access to reproductive health services by street youths is that they will be more exposed to sexual reproductive health issues most especially, sexually transmitted diseases. This has a ripple effect that can lead to deleterious health, economic and social consequences. Furthermore, the rate of unwanted pregnancies will also be on a rise which can invariable lead to clandestine and unsafe abortion. A special attention need to be given to this vulnerable set of youth by the government in order to enhance their overall quality of life.

## Limitation

Extracting intimate sexual information from the street youth was not too easy, however, stressing that confidentiality will be ensured and maintained made data collection possible.

## Conclusion

This study revealed that a larger percentage of street youths were from a polygamous family and most of them had been on the street for more than three months. Two third of respondents had had sex before of which more than one third of them have more than four sexual partners.

Duration on the street was statistically associated with their risky sexual exposure. The Government should create more rehabilitative centers to reduce the length of time they spend on the street. This will invariably shield them from exposure to unwanted pregnancies, sexually transmitted infection as well as unsafe abortion. Also, more enlightenments should be provided by the Government via mass media to discourage street youths from risky sexual behaviors.
